# Distinct Effectiveness of Oritavancin against Tolerance-Induced *Staphylococcus aureus*

**DOI:** 10.3390/antibiotics9110789

**Published:** 2020-11-08

**Authors:** Andrew D. Berti, Lauren T. Harven, Victoria Bingley

**Affiliations:** 1 Department of Pharmacy Practice, Wayne State University College of Pharmacy and Health Sciences, Detroit, MI 48201, USA; gh8899@wayne.edu (L.T.H.); gw2675@wayne.edu (V.B.); 2 Department of Biochemistry, Microbiology and Immunology, Wayne State University College of Medicine, Detroit, MI 48201, USA

**Keywords:** MRSA, oritavancin, tolerance, persistence, lipoglycopeptide

## Abstract

Within a sufficiently large bacterial population, some members will naturally adopt an alternate, metabolically-active state that favors small molecule synthesis over cell division. These isogenic “tolerant” subpopulations have variable responses during antibiotic exposure and can remain viable in the presence of typically bactericidal concentrations. In this study, we determine the ability of typical and atypical antistaphylococcal therapies to reduce the viability of mupirocin-induced tolerant *Staphylococcus aureus* bacteria. Overall, tolerance-induced staphylococci exhibited a markedly decreased rate and extent of killing following antibiotic exposure. However, oritavancin remained effective at maintaining a similar extent of killing. Further studies to investigate the role of oritavancin against recurrent or relapse staphylococcal infection are warranted.

## 1. Introduction

*Staphylococcus aureus* is the most common invasive human pathogen and remains a major contributor to infection-related morbidity and mortality [1]. Clinicians have long recognized the role of antibiotic resistance in treatment failures [2]. Over the past decade, it has become clear that antibiotic tolerance can also contribute to unfavorable clinical outcomes [3]. Antimicrobial tolerance occurs when a small fraction of a bacterial population ceases to focus on cell division and shifts to an alternate metabolic program favoring small molecule synthesis over growth [4,5]. In *S. aureus*, this process can be sharply accelerated by multiple factors present during infection including nutrient limitation, host cationic peptide exposure and polymorphonuclear neutrophil internalization [6,7]. Antimicrobial agents in clinical use are typically most effective against rapidly dividing bacteria, allowing this isogenic subpopulation to survive in otherwise bactericidal concentrations. Survivors of the antibiotic exposure can restart cell division upon cessation of antibiotics and cause relapse or recurrent infection. In this study we determine the ability of typical and atypical antistaphylococcal therapies to reduce the viability of tolerant *Staphylococcus aureus* bacteria.

At the turn of the millennium, vancomycin (VAN) was considered the “antibiotic of last resort” for treatment of methicillin-resistant *Staphylococcus aureus* (MRSA) infection. Since then several antimicrobial agents with anti-MRSA activity have been introduced into clinical practice including daptomycin (DAP), telavancin (TLV), ceftaroline (CPT), dalbavancin (DAL) and oritavancin (ORI). While vancomycin remains the mainstay of contemporary anti-MRSA pharmacotherapy, each of the newer agents has seen use in bloodstream infections, particularly those that do not respond appropriately to vancomycin. Because of their “second-line” clinical utilization, it becomes important to assess how these newer agents perform against antibiotic-tolerant staphylococci. Historically, the infrequent and stochastic behavior of antimicrobial tolerance has limited evaluation of antimicrobials against antibiotic-tolerant subpopulations. However, with the finding that mupirocin exposure can synchronize an entire population into a tolerant state [8,9], we now have the capacity to specifically assess the capability of antimicrobials to reduce viability of tolerant bacteria. Therefore, our primary experimental endpoint is sustained bactericidal activity of an antibiotic over 48 h against a culture synchronized to a tolerant state by high-dose mupirocin exposure. Our secondary endpoint is the minimal duration of exposure sufficient to reduce viability by 99.9% (MDK).

## 2. Results

**Figure 1:**
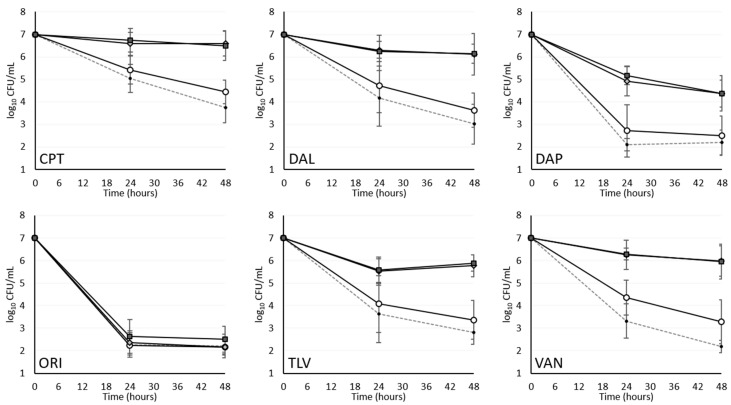
Activity of study antibiotics against mupirocin-induced tolerant staphylococci. Data represent the mean and standard deviation values from five distinct strains, each evaluated in triplicate. Dashed lines, uninduced control; open circles, low mupirocin induction (0.032 µg/mL); gray diamonds, moderate mupirocin induction (0.32 µg/mL); black squares, high mupirocin induction (3.2 µg/mL). Antibiotic abbreviations are as follows: CPT, ceftaroline; DAL, dalbavancin; DAP, daptomycin; ORI, oritavancin; TLV, telavancin; VAN, vancomycin. Uninduced and low-induction exposures were not significantly different at any time point with the exception of CPT and VAN where the two exposures each differed at 48 h (*p <* 0.01). Moderate and high mupirocin induction were essentially indistinguishable from each other and significantly different from uninduced strains (*p <* 0.01) at all time points and for all exposures with the exception of ORI (*p >* 0.05).

As expected, VAN, CPT, DAL, TLV, DAP and ORI reduced the viability of traditional bacterial cultures, frequently achieving bactericidal activity within 48 h (Figure 1). At a low mupirocin concentration, statistically-significant prolongations were observed in the MDK for VAN, CPT, DAL, TLV and DAP although bactericidal activity in general was maintained. The exception to this trend was ORI which demonstrated no significant MDK prolongation (Table 1). At high mupirocin exposure, pronounced prolongations were again observed in the MDK, effectively eliminating the 48 h bactericidal activity of DAP against three isolates and of DAL, TLV, CPT and VAN against all five isolates. In contrast, ORI was the only agent to meet our primary endpoint and maintain bactericidal activity against all isolates at maximal induction of tolerance. We do note, however, the biphasic kill kinetics with ORI resulting in a prolonged MDK for two of the isolates (Table 2).

## 3. Discussion

Antimicrobial tolerance is associated with persistent MRSA endovascular infection and poor clinical outcome [3]. While changes in multiple genes can contribute to enhanced tolerance in staphylococci [10], the associated pathways typically converge on purine biosynthesis [11,12] and the stringent response [6]. This study examined the activity of antistaphylococcal therapies against clinical isolates synchronized to exhibit a stringent response as a consequence of mupirocin exposure.

This is not the first study to examine the impact of antistaphylococcal therapies on nondividing *S. aureus*. Preclinical studies of ORI noted that the agent retained activity against stationary phase cells maintained in nutrient-depleted media, a condition which can induce the stringent response in a stochastic manner [13]. Belley et al. later demonstrated activity of ORI, but not DAL or VAN, against stationary phase (non-dividing) MRSA [14]. Of note, the authors propose the differential activity between DAL and ORI is due to the latter’s membrane depolarization effects. However, our findings do not support this mechanism. DAP, a membrane-active antibiotic with membrane depolarization as a primary mechanism of action, exhibits a marked decrease in DAP killing against tolerant microbes whereas ORI does not. Furthermore, TLV and ORI share the same 4′-chlorobiphenylmethyl hydrophobic group responsible for membrane intercalation and disruption but TLV does not maintain activity against tolerant staphylococci. Alternative mechanisms, such as more effective intercalation of ORI due to its primary binding to pentaglycyl bridges or specific disruption and delocalization of essential membrane proteins should be explored.

One strength of this study is the synchronized induction of the stringent response. Alternative methods such as nutrient deprivation, metabolic arrest or analysis of stationary phase bacteria fail to recapitulate the metabolic activity of tolerant staphylococci and induce only stochastic expression of the tolerant state [15]. Our study is limited by in vitro analysis only in the absence of innate and adaptive host immune responses. Antibiotics were added at a static concentration, although the prolonged half-lives of ORI, DAL and TLV mitigate the influence of antimicrobial pharmacokinetics on our observations. Important staphylococcal lineages such as ST8/USA300 were not represented in the sequential patient isolates assessed in this study. However, tolerance induction in these lineages is a challenge due to their proclivity to carry the pUSA03 plasmid-borne *ileS* gene conferring mupirocin resistance [16]. We do note that during method development we used strain JE2 in our exploratory testing. Strain JE2 is an ST8/USA300 isolate that lacks the variant *ileS* gene. In these experiments, mupirocin supplementation extended the MDK for DAP but not for ORI, suggesting that ST8/USA300 isolates would not respond differently from other lineages (data not shown).

Work is ongoing in our lab to characterize the prevalence and significance of antimicrobial tolerance in clinical isolates. Assessment of clinical outcomes in patients with recurrent staphylococcal infection treated with oritavancin is warranted.

## 4. Materials and Methods

### 4.1. Strain Characterization, Cultivation Conditions and Antibiotic Selection

Strains assessed in this study include the prototypical MSSA strain ATCC 29213 as well as four sequential clinical isolates from patients seen at the Detroit Medical Center. Relevant strain details are presented in Table 3. Antibiotics were supplemented at static concentrations corresponding to the estimated free serum concentration following standard dosing including ceftaroline (CPT, Allergan, Madison, NJ, USA, 17 mg/L), dalbavancin (DAL, Durata Therapeutics, Chicago, IL, USA, 6 mg/L), daptomycin (DAP, Mylan, Canonsburg, PA, USA, 6 mg/L), oritavancin (ORI, Melinta, New Haven CT, USA, 14 mg/L), telavancin (TLV, Theravance Biopharma, San Francisco, CA, USA, 10 mg/L) and vancomycin (VAN, Mylan, New Haven, CT, USA, 35 mg/L). Mupirocin was obtained from Panreac AppliChem, Chicago, IL, USA. Active ceftaroline was generated from ceftaroline fosamil by enzymatic amino dephosphorylation [17] and its activity validated by bioassay prior to use. All media containing DAL, ORI and TLV were supplemented with Tween20 (0.002%) and all media containing DAP were supplemented to 50 mg/L Ca^2+^ per CLSI recommendations [18].

### 4.2. Study Design

Five representative *S. aureus* strains were cultivated in vitro, synchronized in a tolerant state by mupirocin exposure and challenged with antibiotics over a two-day period. Bacterial viability was assessed at predefined time points and the capacity of antibiotic challenge to reduce culture viability determined.

### 4.3. Induction of Antimicrobial Tolerance

Bacteria were cultured overnight in Mueller–Hinton Broth (37 °C, 180 rpm). Overnight cell cultures were normalized to a McFarland standard turbidity of 0.5 corresponding to approximately 1 × 10^8^ colony forming units (cfu) per mL. Four replicates of the standard suspension were diluted in fresh medium to an inoculum of 10^6^ cfu/mL, supplemented with mupirocin (0, 0.032, 0.32, 3.2 mg/L) and cultured with shaking for 1 h (37 °C, 180 rpm) after which antibiotics were added at the indicated concentrations. Samples were removed for colony enumeration immediately prior to addition of antibiotics and at set intervals after antibiotic challenge. Samples were enumerated by dilution plating on brain heart infusion agar. Antibiotics were considered “bactericidal” if the viable cfu/mL decreased from baseline by more than 3 log_10_ units over a 48 h period. The minimum duration to bactericidal activity (MDK_99.9_) [5] was determined individually per replicate via linear extrapolation between the timepoints immediately preceding and following a 3 log_10_ unit reduction from baseline. All analyses were performed in triplicate.

### 4.4. Statistical Analysis

Descriptive data was expressed as mean and standard deviation, median and interquartile range, or frequencies and percentage. Univariable analysis was performed using a Student’s *t*-test, Wilcoxon rank sum, or Fisher’s Exact test for continuous, ordinal, and categorical data, respectively.

## 5. Conclusions

Oritavancin retains bactericidal activity in vitro against tolerant *S. aureus*, whereas alternative antistaphylococcal antibiotics do not. Oritavancin should be considered for treatment of recurrent *S. aureus* infection where antimicrobial tolerance is suspected or confirmed.

## Figures and Tables

**Table 1:** Time to bactericidal activity under low induction conditions. Under low mupirocin exposure, MDK is prolonged in an isolate- and antibiotic-dependent manner. Dashed line (—), MDK not achieved over 48 h; *, *p <* 0.05.

Strain Name	MDK (Uninduced, h)						MDK (Low Induction, h)					
	*CPT*	*DAL*	*DAP*	*ORI*	*TLV*	*VAN*	*CPT*	*DAL*	*DAP*	*ORI*	*TLV*	*VAN*
29213	—	19 ± 0.6	1 ± 0.0	1 ± 0.0	37 ± 0.4	13 ± 0.6	—	24 ± 0.1 *	47 ± 1.5 *	2 ± 0.5	48 ± 0.0 *	21 ± 1.0 *
BSN10	39 ± 0.8	—	2 ± 0.0	1 ± 0.1	38 ± 5.9	22 ± 0.4	—	—	13 ± 1.1 *	1 ± 0.2	44 ± 6.1	—
BSN11	28 ± 1.0	35 ± 4.0	1 ± 0.0	1 ± 0.0	14 ± 0.2	18 ± 3.9	36 ± 11.0	46 ± 2.9 *	1 ± 0.1	1 ± 0.0	44 ± 6.4 *	37 ± 9.6
BSN12	—	34 ± 3.5	1 ± 0.0	1 ± 0.0	17 ± 4.7	20 ± 0.6	—	40 ± 4.5	2 ± 0.0 *	1 ± 0.0	13 ± 2.9	36 ± 2.7 *
BSN13	39 ± 5.1	17 ± 0.8	1 ± 0.0	1 ± 0.0	19 ± 1.4	24 ± 5.5	47 ± 1.3	18 ± 1.0	1 ± 0.1	1 ± 0.0	22 ± 6.0	26 ± 8.2

**Table 2:** Time to bactericidal activity under high induction conditions. Under high mupirocin exposure, MDK is prolonged or not achieved for most isolates and antibiotics. Dashed line (—), MDK not achieved over 48 h; *, *p <* 0.05.

Strain Name	MDK (Uninduced, h)						MDK (High Induction, h)					
	*CPT*	*DAL*	*DAP*	*ORI*	*TLV*	*VAN*	*CPT*	*DAL*	*DAP*	*ORI*	*TLV*	*VAN*
29213	—	19 ± 0.6	1 ± 0.0	1 ± 0.0	37 ± 0.4	13 ± 0.6	—	—	—	5 ± 0.4 *	—	—
BSN10	39 ± 0.8	—	2 ± 0.0	1 ± 0.1	38 ± 5.9	22 ± 0.4	—	—	—	27 ± 6.5 *	—	—
BSN11	28 ± 1.0	35 ± 4.0	1 ± 0.0	1 ± 0.0	14 ± 0.2	18 ± 3.9	—	—	40 ± 2.2 *	1 ± 0.1	—	—
BSN12	—	34 ± 3.5	1 ± 0.0	1 ± 0.0	17 ± 4.7	20 ± 0.6	—	—	—	4 ± 1.6	—	—
BSN13	39 ± 5.1	17 ± 0.8	1 ± 0.0	1 ± 0.0	19 ± 1.4	24 ± 5.5	—	—	41 ± 10.2 *	2 ± 0.9	—	—

**Table 3:** Study strains. Minimum inhibitory concentrations were determined by Etest (Biomérieux) or broth microdilution consistent with CLSI standards [18]. BSN strains are patient isolates obtained from consecutive patients presenting to the Detroit Medical Center with staphylococcal bacteremia. ATCC, the American Type Culture Collection, Manassas, VA.

Strain Name	Source	Genetic Characterization	Minimum Inhibitory Concentration (mg/L)					
			CPT	DAL	DAP	ORI	TLV	VAN
29213	ATCC	ST5-MSSA *spa* t010 *agr2*	0.25	0.06	0.25	0.06	0.06	1.0
BSN10	This Study	ST45-MSSA *spa* t065 *agr1*	0.19	0.03	0.19	0.06	0.13	1.0
BSN11	This Study	ST15-MSSA *spa* t10135 *agr2*	0.25	0.03	0.25	0.13	0.06	1.0
BSN12	This Study	ST5-MRSA-IVg *spa* t688 *agr2*	0.38	0.03	0.13	0.06	0.06	1.0
BSN13	This Study	ST97-MSSA *spa* t224 *agr1*	0.19	0.05	0.13	0.13	0.13	1.0
